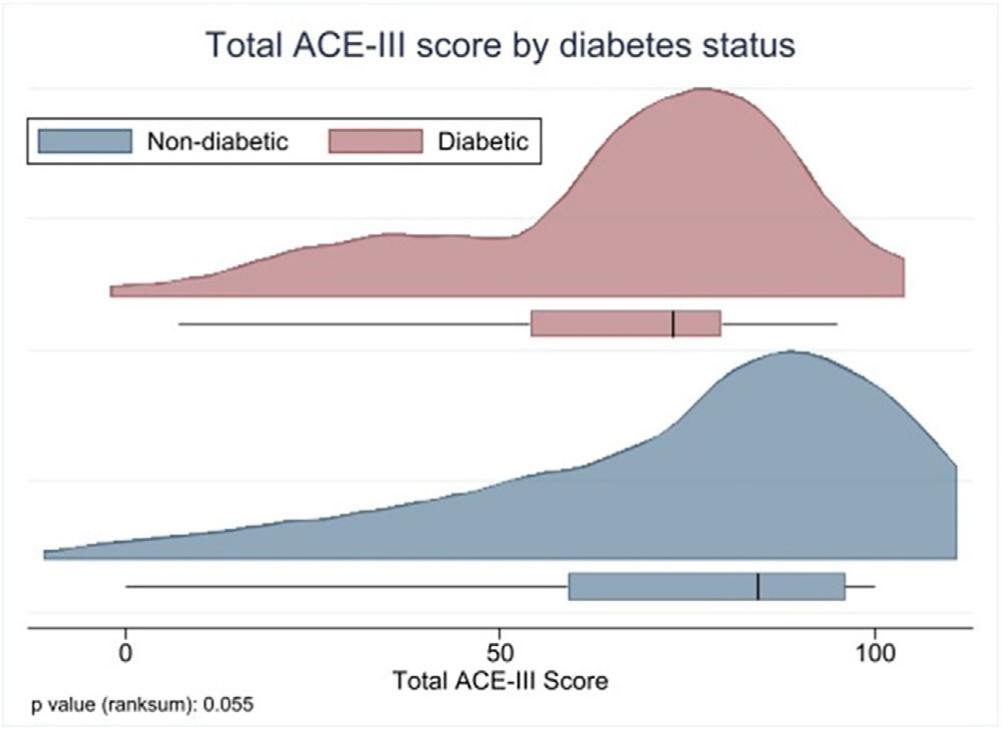# Brain health in Diabetic Seniors: Understanding the Dementia dynamics

**DOI:** 10.1002/alz.094719

**Published:** 2025-01-09

**Authors:** Ambica Singh, Aparajit Ballav Dey, Vignesh Dwarakanathan

**Affiliations:** ^1^ All India Institute of Medical Sciences, New Delhi, New Delhi, Delhi India; ^2^ Venu Geriatric Institute, South Delhi, Delhi India; ^3^ ESIC Medical College and Hospital, Chennai, Tamil Nadu India

## Abstract

**Background:**

In a landscape where both cognitive decline and diabetes are on the ascent globally, an increasingly pertinent question emerges: what interconnections exist between dementia and diabetes in older individuals? Cognitive impairment is a decline in mental abilities that affects memory, attention, reasoning, and other cognitive functions leading to dementia. Diabetes is associated with an increased risk of cognitive impairment and dementia, as well as vascular complications that damage the brain. Several studies have shown that type 2 diabetes and hypertension can impair blood‐brain barrier integrity, cerebral circulation, glucose metabolism, inflammation, oxidative stress, and amyloid‐beta production in the brain. Here, we endeavor to find any correlation between stages of cognitive decline and diabetes in the elderly for better insight and enhanced geriatric care.

**Method:**

In this comparative cross‐sectional study, patients aged 60 years and beyond were recruited from Memory Clinic, Department of Geriatric Medicine, AIIMS, New Delhi. Based on Addenbrooke’s Cognitive Examination‐III (ACE‐III) scoring for cognition, patients with cognitive decline were divided into 3 categories: Subjective Cognitive Impairment (SCI), Mild Cognitive Impairment (MCI) and Major Neurocognitive Disorder (MNCD). 20 patients in each category were taken against 20 controls. Diabetic status was self‐reported and as per their HbA1c levels.

**Result:**

Significant cognitive decline was found in patients with type 2 diabetes with a p‐value of 0.02. As against controls, the association was found highest in MCI group, while SCI had the lowest percentage of diabetics. ACE‐III scores were significantly affected in diabetics. The most affected domain was fluency (p‐value 0.03) followed by memory (p‐value 0.12).

**Conclusion:**

This study shows a clear correlation between cognitive decline and diabetic status in older patients. This work has translational value and clinical utility in the future.